# A Subset of Autism-Associated Genes Regulate the Structural Stability of Neurons

**DOI:** 10.3389/fncel.2016.00263

**Published:** 2016-11-17

**Authors:** Yu-Chih Lin, Jeannine A. Frei, Michaela B. C. Kilander, Wenjuan Shen, Gene J. Blatt

**Affiliations:** ^1^Laboratory of Neuronal Connectivity, Program in Neuroscience, Hussman Institute for Autism, BaltimoreMD, USA; ^2^Laboratory of Autism Neurocircuitry, Program in Neuroscience, Hussman Institute for Autism, BaltimoreMD, USA

**Keywords:** autism-risk genes, neurite outgrowth, dendrite, dendritic spine, synapse formation, actin, adhesion molecule

## Abstract

Autism spectrum disorder (ASD) comprises a range of neurological conditions that affect individuals’ ability to communicate and interact with others. People with ASD often exhibit marked qualitative difficulties in social interaction, communication, and behavior. Alterations in neurite arborization and dendritic spine morphology, including size, shape, and number, are hallmarks of almost all neurological conditions, including ASD. As experimental evidence emerges in recent years, it becomes clear that although there is broad heterogeneity of identified autism risk genes, many of them converge into similar cellular pathways, including those regulating neurite outgrowth, synapse formation and spine stability, and synaptic plasticity. These mechanisms together regulate the structural stability of neurons and are vulnerable targets in ASD. In this review, we discuss the current understanding of those autism risk genes that affect the structural connectivity of neurons. We sub-categorize them into (1) cytoskeletal regulators, e.g., motors and small RhoGTPase regulators; (2) adhesion molecules, e.g., cadherins, NCAM, and neurexin superfamily; (3) cell surface receptors, e.g., glutamatergic receptors and receptor tyrosine kinases; (4) signaling molecules, e.g., protein kinases and phosphatases; and (5) synaptic proteins, e.g., vesicle and scaffolding proteins. Although the roles of some of these genes in maintaining neuronal structural stability are well studied, how mutations contribute to the autism phenotype is still largely unknown. Investigating whether and how the neuronal structure and function are affected when these genes are mutated will provide insights toward developing effective interventions aimed at improving the lives of people with autism and their families.

## Introduction

Autism spectrum disorder (ASD) is a neurodevelopmental clinical condition currently diagnosed based on the American Psychiatric Association’s Diagnostic and Statistical Manual of Mental Disorders, Fifth Edition (DSM-5) criteria reflecting symptoms, possibly of varying severity, in social interaction, communication and behavior (American Psychiatric Association, 2013; Lord and Jones, 2013). ASD occurs in 1:68 individuals in the United States (Baio, 2014) and complex genetic interactions appear responsible for a high degree of heterogeneity of the clinical symptoms in ASD. Individuals with ASD often co-express other comorbidities including epilepsy which often complicates diagnosis and treatment. Alterations in neuronal structures in different brain regions have been reported in ASD individuals, including increased dendritic spine density in cortical pyramidal neurons (Hutsler and Zhang, 2010; Tang et al., 2014) as well as stunting of dendritic branching in the hippocampus (Raymond et al., 1996; Bauman and Kemper, 2005). In addition, subcortical band heterotopia, representing alterations in cell migration has also been found in a child with ASD (Beaudoin et al., 2007). These brain regions are often characterized with neuroanatomical irregularities in ASD (Donovan and Basson, 2016). The defective regulation for structural stability of neurons may be one of the underlying mechanisms that contribute to the anatomical changes in ASD.

**FIGURE 1:**
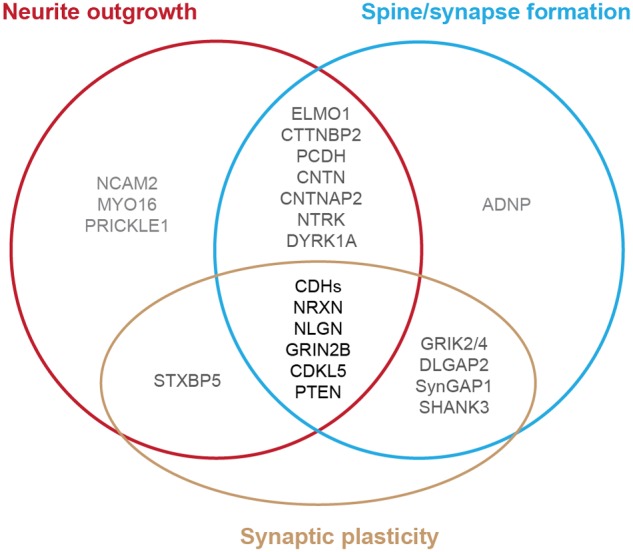
**Diagram of autism-risk genes implicated in regulating the structural stability of neurons.** Each circle represents a cellular pathway to regulate the structural stability of neurons, including neurite outgrowth (red), dendritic spine or synapse formation (blue), and synaptic plasticity (gold). Experimental evidence shows that many autism-risk genes regulate at least one cellular pathway to maintain the integrity of neuronal structures. Genes that regulate only one pathway are labeled in light gray. Genes that regulate two pathways are labeled in dark gray. Genes that regulate three pathways are labeled in black. The summaries of autism-risk genes that affect each cellular pathway can be found in **Tables 1**–**3**.

Autism spectrum disorder is typically diagnosed during the first 3 years of life, a period of extensive neurite formation, synaptogenesis and refinement (Huttenlocher and Dabholkar, 1997; Zoghbi and Bear, 2012; Stamou et al., 2013; McGee et al., 2014). Indeed, brain imaging studies from individuals with ASD and anatomical measurements of neuronal structure in post-mortem tissues exhibit differences in neuronal connectivity derived from the disruption of neurite outgrowth, synapse formation and stabilization (Raymond et al., 1996; Hutsler and Zhang, 2010; Penzes et al., 2011). Studies of human induced pluripotent stem cells (iPSCs) derived from people with ASD also have identified defects of neuronal structure (Habela et al., 2015; Nestor et al., 2015). Genome-wide association studies on individuals with ASD and their families revealed several risk genes that may be the common molecular targets in autism (Bucan et al., 2009; Glessner et al., 2009; Hussman et al., 2011; O’Roak et al., 2011, 2012a; Buxbaum et al., 2012, 2014; Sanders et al., 2012; Shi et al., 2013; Stamou et al., 2013; Yu et al., 2013; An et al., 2014; Brett et al., 2014; Cukier et al., 2014; De Rubeis et al., 2014; Iossifov et al., 2014; McGee et al., 2014; Pinto et al., 2014; Ronemus et al., 2014; Toma et al., 2014; Yuen et al., 2015). Animal studies of these genes further identify several specific cellular pathways during brain development that are vulnerable in ASD, including the disruption of neurite outgrowth, dendritic spine formation, and synaptic function (**Figure 1**) (Walsh et al., 2008; Bourgeron, 2009; Hussman et al., 2011; Penzes et al., 2011; Zoghbi and Bear, 2012; Ebert and Greenberg, 2013; Stamou et al., 2013; Bernardinelli et al., 2014; De Rubeis et al., 2014; Pinto et al., 2014; Phillips and Pozzo-Miller, 2015). Differences in environment as well as the presence of multiple gene mutations occurring in the same individual with autism complicate studies of the relationship between each gene and the phenotype observed. However, because similar cellular pathways (e.g., neurite outgrowth) are altered in different affected individuals, we can potentially develop therapeutic interventions to help mitigate the autism phenotypes.

During development, neurite outgrowth and synapse formation are dynamic processes and their maturation is mutually dependent on proper guidance. Neurites initially exhibit frequent branch additions and retractions. Once dendrite arbors are established, productive synapse formation later in life and the accompanying activation of post-synaptic signaling machinery promotes arbor stability (Dailey and Smith, 1996; Wu and Cline, 1998; Rajan et al., 1999; Wong et al., 2000; Cline, 2001; Niell et al., 2004). Conversely, a loss of synaptic inputs leads to dendritic loss (Jones and Thomas, 1962; Matthews and Powell, 1962; Coleman and Riesen, 1968; Sfakianos et al., 2007). This reciprocal regulation contributes to the refinement of dendrites and synapses as the neurons mature (Wu et al., 1999; Trachtenberg et al., 2002; Holtmaat et al., 2005; Koleske, 2013). Thus, maintaining the structural stability of neurons and synapses is critical for proper brain function. Alterations in these processes likely underlie the disruption of normal dendrite and dendritic spine structure in neurological disorders, including neurodevelopmental conditions, psychiatric disorders, and neurodegenerative diseases (Fiala et al., 2002; Lin and Koleske 2010; Penzes et al., 2011; Kulkarni and Firestein, 2012; Zoghbi and Bear, 2012; Koleske, 2013; Bernardinelli et al., 2014).

**FIGURE 2:**
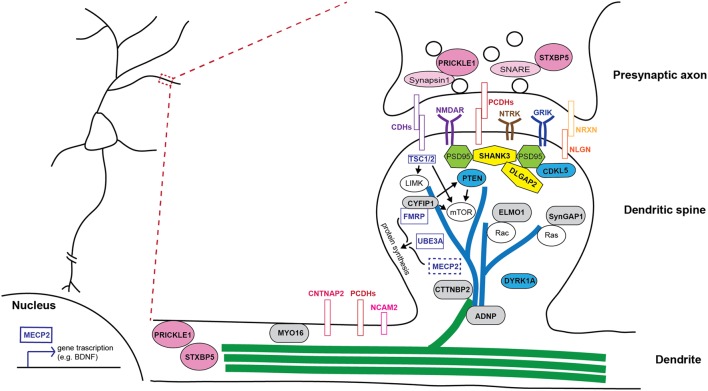
**Schematic illustration of how autism-risk genes regulate neuronal structure and their sites of action.** An illustration of a dendritic segment containing a dendrite and a dendritic spine is enlarged from the box region on the left and shown on the right. Microtubules (green) and actin filaments (blue) are two major cytoskeletons found in dendrites and dendritic spines, respectively. Autism-risk genes (in bold font) are categorized by their main function and color coded accordingly. (1) Cytoskeletal proteins (gray rounded rectangular box): MYO16, CTTNBP2, and ADNP, directly regulate actin and microtubule function to control dendritic spine and neurite stability. ELMO1 and SYNGAP1 regulate actin dynamics to control spine stability via small RhoGTPases. (2) Adhesion molecules (colored rectangular box): Cadherins (CDHs), protocadherins (PCDHs), and neurexin (NRXN)-neuroligin (NLGN) complex, as well as surface receptors, NTRK, GRIK, and NMDAR, act at synapses to regulate synaptic function. NCAM2 and CNTNAP2, also have functions in regulating neurite outgrowth. (3) Signaling molecules (blue ellipse shape): CDKL5, DYRK1A, and PTEN regulate several signaling pathways to maintain the stability of dendritic structures. (4) Scaffolding proteins (yellow polygon): SHANK3 and DLGAP2, locate at post-synaptic density and tightly associate with PSD95 and other signaling molecules to regulate spine stability and synaptic plasticity. (5) Synaptic proteins (pink ellipse shape): STXBP5 and PRICKLE1 not only regulate synaptic vesicle release, but also play a role in regulating neurite outgrowth. (6) Syndromic molecules (clear rectangular box): FMRP and UBE3A regulate the structural stability of neurons via the regulation of protein synthesis or binding with other molecules in dendritic spines. MECP2 mainly functions in the nucleus and regulates transcription of many genes to in turn affecting the structural stability of neurons. TSC1/2 regulates the mTOR pathway and cytoskeletal machinery to maintain dendritic stability.

It is well-accepted that ASD is not a monogenetic disorder, instead, it is often a neurological condition resulted from multiple mutations of several different genes. Although knockout, knockin, or transgenic approaches of autism-risk genes in animal models have demonstrated some of the autistic-like behaviors (Kazdoba et al., 2016), the limitation of the number of genes being manipulated in animals makes it difficult to recapitulate the human condition experimentally. Furthermore, ASD is a common comorbid condition in individuals with other neurodevelopmental disorders. The similar representation of the symptoms but different contribution of genetic mutations often complicates the diagnosis and the treatment. The complex profile of gene mutations makes it difficult to call a gene “the autism gene.” However, the list of autism-risk genes provides us a direction to understand the potentially vulnerable pathways in neurons that may be therapeutic targets to develop more efficient interventions for ASD. Indeed, in addition to the structural stability of neurons, several cellular pathways including transcriptional regulation (De Rubeis et al., 2014; Sanders, 2015), excitatory/inhibitory (E/I) balance (Blatt et al., 2001; Hussman, 2001; Rubenstein and Merzenich, 2003; Gao and Penzes, 2015; Nelson and Valakh, 2015), cerebellar development (Wang et al., 2014; Hampson and Blatt, 2015), and autoregulatory feedback loops (Mullins et al., 2016) have been proposed to be vulnerable in autism. In this review, we focus on recent identified autism-risk genes that have been shown to regulate neuronal structures and circuit formation, including aspects of neurite outgrowth (**Table 1**), synapse formation and spine stability (**Table 2**), and synaptic plasticity (**Table 3**). We will discuss the known biological function of those individual autism-risk genes in neurons and how they converge into common pathways. We have categorized these genes into cytoskeletal regulators, adhesion molecules, cell surface receptors, signaling molecules, as well as synaptic proteins (**Figure 2**). In addition, we include genes causing syndromic disorders in the discussion to highlight the importance of maintaining the neuronal structures for proper brain function.

**Table 1:** Summary of autism-associated genes that regulate neurite outgrowth.

Functional category	Gene	Representative references
**Cytoskeletal regulator**		
	*MYO16*	Patel et al., 2001; Yokoyama et al., 2011
	*CTTNBP2*	Shih et al., 2014
	*ELMO1*	Franke et al., 2012; Lanoue et al., 2013
**Adhesion molecule**		
	*CDHs*	Esch et al., 2000; Bekirov et al., 2008; Tan et al., 2010; Friedman et al., 2015b
	*PCDH*	Uemura et al., 2007; Morrow et al., 2008; Keeler et al., 2015
	*NRXN*	Gjorlund et al., 2012
	*NLGN*	Gjorlund et al., 2012
	*CNTNAP2*	Anderson et al., 2012
	*CNTN*	Ye et al., 2008
	*NCAM2*	Sheng et al., 2015
**Surface receptor**		
	*GRIN2B*	Ewald et al., 2008; Espinosa et al., 2009; Sepulveda et al., 2010; Bustos et al., 2014
	*NTRK*	Joo et al., 2014
**Signaling molecule**		
	*DYRK1A*	Hammerle et al., 2003; Benavides-Piccione et al., 2005; Gockler et al., 2009; Lepagnol-Bestel et al., 2009
	*CDKL5*	Chen et al., 2010b; Amendola et al., 2014; Fuchs et al., 2014
	*PTEN*	Jaworski et al., 2005; Kwon et al., 2006; Zhou et al., 2009
**Synaptic protein**		
	*STXBP5*	Sakisaka et al., 2004
	*PRICKLE1*	Liu et al., 2013a
**Syndromic disorder related gene**		
	*FMR1*	Galvez et al., 2003; Antar et al., 2006; Tucker et al., 2006; Berman et al., 2012; Amiri et al., 2014
	*MECP2*	Fukuda et al., 2005; Jugloff et al., 2005; Zhou et al., 2006; Ballas et al., 2009; Belichenko et al., 2009; Kishi and Macklis, 2010; Cohen et al., 2011; Marshak et al., 2012; Nguyen et al., 2012; Stuss et al., 2012; Jiang et al., 2013a; Baj et al., 2014
	*UBE3A*	Dindot et al., 2008; Miao et al., 2013; Valluy et al., 2015
	*TSC1/2*	Floricel et al., 2007; Choi et al., 2008


**Table 2:** Summary of autism-associated genes that regulate synapse/spine formation.

Functional category	Gene	Representative references
**Cytoskeletal regulator**		
	*ADNP*	Oz et al., 2014
	*CTTNBP2*	Chen and Hsueh, 2012; Chen et al., 2012; Hsueh, 2012
	*SYNGAP1*	Chen et al., 1998; Krapivinsky et al., 2004; Oh et al., 2004; Rumbaugh et al., 2006; Clement et al., 2012; Aceti et al., 2015
	*ELMO1*	Kim et al., 2011a
**Adhesion molecule**		
	*CDHs*	Benson and Tanaka, 1998; Huntley and Benson, 1999; Bozdagi et al., 2000; Togashi et al., 2002; Paradis et al., 2007; Suzuki et al., 2007; Bekirov et al., 2008; Williams et al., 2011; Friedman et al., 2015b
	*PCDH*	Tsai et al., 2012a
	*NRXN*	de Wit et al., 2009; Ko et al., 2009; Gokce and Sudhof, 2013
	*NLGN*	Scheiffele et al., 2000; Prange et al., 2004; Chih et al., 2005; Levinson et al., 2005; Varoqueaux et al., 2006; Kwon et al., 2012;
	*CNTNAP2*	Anderson et al., 2012; Gdalyahu et al., 2015; Varea et al., 2015
	*CNTN*	Li et al., 2003; Takeda et al., 2003; Sakurai et al., 2009, 2010; Toyoshima et al. 2009
**Surface receptor**		
	*GRIN2B*	Akashi et al., 2009; Espinosa et al., 2009; Brigman et al., 2010; Ohno et al., 2010; Kelsch et al., 2012
	*GRIK2/4*	Huettner, 2003; Lanore et al., 2012
	*NTRK*	Menn et al., 2000
**Signaling molecule**		
	*DYRK1A*	Benavides-Piccione et al., 2005; Park et al., 2012; Thomazeau et al., 2014
	*CDKL5*	Zhu et al., 2013; Della Sala et al., 2016
	*PTEN*	Kwon et al., 2006; Fraser et al., 2008; Luikart et al., 2011; Pun et al., 2012; Zhang et al., 2012; Haws et al., 2014; Cupolillo et al., 2016
**Synaptic protein**		
	*SHANK3*	Sala et al., 2001; Roussignol et al., 2005; Hung et al., 2008; Peça et al., 2011; Verpelli et al., 2011; Wang et al., 2011b; Peixoto et al., 2016
	*DLGAP2*	Jiang-Xie et al., 2014
**Syndromic disorder related gene**		
	*FMR1*	Comery et al., 1997; Weiler and Greenough, 1999; Braun and Segal, 2000; Irwin et al., 2000; Nimchinsky et al., 2001; Segal et al., 2003; Galvez and Greenough, 2005; Koekkoek et al., 2005; McKinney et al., 2005; Antar et al., 2006; Grossman et al., 2006, 2010; Dictenberg et al., 2008; Cruz-Martin et al., 2010; Pan et al., 2010; Levenga et al., 2011a; Qin et al., 2011; Berman et al., 2012; He and Portera-Cailliau, 2013; Amiri et al., 2014; Wijetunge et al., 2014
	*MECP2*	Fukuda et al., 2005; Zhou et al., 2006; Chapleau et al., 2009; Nguyen et al., 2012; Stuss et al., 2012; Jiang et al., 2013a; Baj et al., 2014
	*UBE3A*	Dindot et al., 2008; Greer et al., 2010; Yi et al., 2015; Kim et al., 2016; Valluy et al., 2015
	*TSC1/2*	Tavazoie et al., 2005


**Table 3:** Summary of autism-associated genes that regulate synaptic plasticity.

Functional category	Gene	Representative references
**Cytoskeletal regulator**		
	*SYNGAP1*	Vazquez et al., 2004; Rumbaugh et al., 2006; Carlisle et al., 2008; Clement et al., 2012, 2013; Ozkan et al., 2014
**Adhesion molecule**		
	*CDHs*	Bozdagi et al., 2000; Manabe et al., 2000; Togashi et al., 2002; Bozdagi et al., 2010; Mendez et al., 2010
	*NRXN*	Levinson et al., 2005; Etherton et al., 2009
	*NLGN*	Chih et al., 2005; Varoqueaux et al., 2006; Chubykin et al., 2007; Tabuchi et al., 2007
**Surface receptor**		
	*GRIN2B*	Brigman et al., 2010; Ohno et al., 2010; Wang et al., 2011a; Yang et al., 2012a; Ryan et al., 2013; Dupuis et al., 2014
	*GRIK2/4*	Contractor et al., 2001; Huettner, 2003; Youn and Randic, 2004; Lanore et al., 2012; Aller et al., 2015
**Signaling molecule**		
	*CDKL5*	Della Sala et al., 2016
	*PTEN*	Fraser et al., 2008; Luikart et al., 2011
**Synaptic protein**		
	*SHANK3*	Bangash et al., 2011; Peça et al., 2011; Wang et al., 2011b; Peixoto et al., 2016
	*DLGAP2*	Jiang-Xie et al., 2014
	*STXBP5*	Barak et al., 2013; Ben-Simon et al., 2015
**Syndromic disorder related gene**		
	*FMR1*	Segal et al., 2003; Koekkoek et al., 2005; Bureau et al., 2008; Auerbach and Bear, 2010
	*MECP2*	Collins et al., 2004; Dani et al., 2005; Asaka et al., 2006; Moretti et al., 2006; Chao et al., 2007; Zhang et al., 2008; Cohen et al., 2011; Li et al., 2011; Noutel et al., 2011; Blackman et al., 2012; Na et al., 2012; Qiu et al., 2012; Zhong et al., 2012; Na et al., 2013; Della Sala and Pizzorusso, 2014; Deng et al., 2014; De Filippis et al., 2015
	*UBE3A*	Yashiro et al., 2009; Sato and Stryker, 2010; Smith et al., 2011; Hayrapetyan et al., 2014


## Cytoskeletal Regulation Is Key to the Maintenance of Proper Stability and Plasticity of Neurons

The actin and microtubule cytoskeletons are the major components of dendritic spine and neurite structure, respectively (Hoogenraad and Akhmanova, 2010; Hotulainen and Hoogenraad, 2010; Dent et al., 2011; Shirao and Gonzalez-Billault, 2013). Precise regulation of these actin and microtubule networks is thus central to guide the proper development, plasticity, and long-term stability of these structures (Matus, 2000; Matus et al., 2000; Luo, 2002; Lin and Webb, 2009; Korobova and Svitkina, 2010; Lin and Koleske, 2010; Svitkina et al., 2010; Dent et al., 2011; Nicholson et al., 2012; Penzes and Cahill, 2012; Penzes and Rafalovich, 2012; Saneyoshi and Hayashi, 2012).

### Actin and Microtubule Regulators Are Associated with Autism

Myosins are motors that utilize ATPase activity to provide motility of actin or cargo transport on actin filaments (Pollard and Korn, 1973; Oliver et al., 1999; Tyska and Warshaw, 2002). Several myosin isoforms play central roles in regulating neurite outgrowth, as well as dendritic spine structural plasticity (Wylie et al., 1998; Wylie and Chantler, 2003; Ryu et al., 2006; Hammer and Wagner, 2013; Kneussel and Wagner, 2013; Yoshii et al., 2013; Koskinen et al., 2014; Ultanir et al., 2014). Among all isoforms, *MYO16* (Myr8 or NYAP3) was recently implicated in ASD (Wang et al., 2009; Connolly et al., 2013; Kenny et al., 2014; Roberts et al., 2014; Liu et al., 2015b). MYO16 is expressed predominantly in the cortex and cerebellum. Levels and phosphorylation of MYO16 protein peak during early developmental stages, consistent with a role in regulating neuronal migration and neurite extension (Patel et al., 2001; Yokoyama et al., 2011). In addition to binding directly to filamentous- (F-)actin, MYO16 also physically interacts with PI3K and WAVE complex to regulate stress fiber remodeling in fibroblasts as well as the adhesion-dependent neurite outgrowth in neurons (Yokoyama et al., 2011).

*CTTNBP2* encodes cortactin-binding protein 2 that interacts with cortactin, a nucleation-promoting factor of actin (Ohoka and Takai, 1998). CTTNBP2 is highly expressed in dendritic spines where it locally interacts with cortactin, striatin, a calcium binding protein, and PP2A, a serine/threonine protein phosphatase 2A, to control the formation and the maintenance of dendritic spines (Chen et al., 2012; Chen and Hsueh, 2012; Hsueh, 2012). In addition, oligomerization of CTTNBP2 induces microtubule bundling to promote dendrite arborization (Shih et al., 2014). Several mutations of *CTTNBP2* have been reported in ASD cases, further indicating the importance of neurite outgrowth and dendritic spine formation for proper brain function (Cheung et al., 2001; Iossifov et al., 2012; De Rubeis et al., 2014).

Activity-dependent neuroprotective protein (ADNP) is a homeobox-containing protein secreted from glia and neurons (Bassan et al., 1999; Zamostiano et al., 2001; Furman et al., 2004; Mandel et al., 2008; Nakamachi et al., 2008). Through its interaction with the chromatin remodeling complex SWI/SNF, ADNP regulates hundreds of genes to modulate brain function (Pinhasov et al., 2003; Mandel and Gozes, 2007; Mandel et al., 2007). In addition to its traditional role in regulating transcription, ADNP has also been suggested to have function in regulating dendritic spines through interactions with microtubule end binding proteins (Oz et al., 2014). Mutations in or the alteration of the protein expression of *ADNP* have been associated with several neurological disorders, including schizophrenia and Alzheimer’s Disease (Vulih-Shultzman et al., 2007; Fernandez-Montesinos et al., 2010; Dresner et al., 2011; Yang et al., 2012b). Intriguingly, the association of mutations in *ADNP* and ASD is further emphasizing that the cytoskeletal integrity of neurons is vulnerable in ASD (O’Roak et al., 2012a,b; Ben-David and Shifman, 2013; De Rubeis et al., 2014; Helsmoortel et al., 2014; Vandeweyer et al., 2014; D’Gama et al., 2015).

### Small RhoGTPase Regulation Is a Key Mechanism in Controlling Neurite and Spine Stability

Small RhoGTPases including Rho, Rac, and Cdc42 are central cytoskeletal regulators that control cell motility and morphology (Govek et al., 2005; Newey et al., 2005; Lin and Koleske, 2010; Tolias et al., 2011). Genetic mutations or dysregulation of the small RhoGTPase regulators, including guanine-exchange factors (GEFs) and GTPase-activating proteins (GAPs), have been implicated in several neurological conditions, including ASD (Newey et al., 2005; Lin and Koleske, 2010; Antoine-Bertrand et al., 2011; Stankiewicz and Linseman, 2014). Here, we will highlight those that regulate the morphological stability of neurons.

Engulfment and cell motility 1 (ELMO1) was first identified in a complex with a RacGEF, DOCK180, to activate Rac1 activity, which is essential for cell migration and phagocytosis (Gumienny et al., 2001; Brugnera et al., 2002; Grimsley et al., 2004). In hippocampal neurons, ELMO1 and DOCK180 colocalize at synaptic sites and together are required for spine formation (Kim et al., 2011a). Loss of *Elmo1* shows a reduction in spine number but increased filopodia, suggesting a role in formation and/or maintenance of mature spines (Kim et al., 2011a). In addition, ELMO1 has been shown to regulate axonal and dendritic branching via Rac1 activation in response to different upstream signals (Franke et al., 2012; Lanoue et al., 2013).

*SYNGAP1* encodes a synaptic-specific Ras/Rap GAP that associates with PSD-95 and specifically localizes to synaptic sites (Kim et al., 1998; Krapivinsky et al., 2004). The role of SYNGAP1 in regulating spine morphology and synaptic function has been well described. In response to CaMKII phosphorylation, SYNGAP1 directly regulates Ras/Rap activity to modulate MAPK signaling to maintain the stability of dendritic spines (Chen et al., 1998; Krapivinsky et al., 2004; Oh et al., 2004; Rumbaugh et al., 2006). Overexpression of SYNGAP1 decreases AMPAR-mediated currents and surface expression (Rumbaugh et al., 2006; Wang et al., 2013). Deletion or reduction of SYNGAP1 results in an elevated synaptic strength and an increase of mushroom spines (Vazquez et al., 2004; Rumbaugh et al., 2006; Carlisle et al., 2008). Mice with *Syngap1* haploinsufficiency show accelerated maturation of dendritic spines followed by disruptions of synaptic transmission and cognitive function (Clement et al., 2012, 2013; Ozkan et al., 2014; Aceti et al., 2015). Coincidentally, haploinsufficiency in *SYNGAP1* has been found in individuals with autism, intellectual disability, and a specific form of epilepsy (Berryer et al., 2013). Several other *de novo* mutations of *SYNGAP1* also have been identified in different cases of ASD (Pinto et al., 2010; Cook, 2011; Hamdan et al., 2011; Berryer et al., 2013; Willsey et al., 2013; Brett et al., 2014; De Rubeis et al., 2014; O’Roak et al., 2014).

Since the actin and microtubule cytoskeletons are the major components of neuronal processes, it is not surprising that manipulating the cytoskeletal machinery dramatically affects neuronal structures. A small imbalance of cytoskeletal dynamics will create a huge impact on the structural stability of neurons, which in turn alters the formation of neuronal circuitry. Interestingly, most autism-associated cytoskeletal regulators control neurite outgrowth and synapse/spine formation thereby affecting the structural stability of neurons. These two processes are also the initial steps to establish correct neuronal connections during development. Failure to regulate these processes properly may result in significantly altered wiring of brain circuitries that is often found in ASD. The next research focus should investigate early in development to connect the dysregulatory effects of mutations in cytoskeletal genes.

## *Trans*-Synaptic Adhesion Molecules Play Important Roles in the Regulation of Neuronal Stability

Cell adhesion molecules (CAMs) play crucial roles in many aspects of neural circuit formation and, thus, it comes as no surprise that these molecules are found as top hits in lists of autism risk genes (Betancur et al., 2009; Pinto et al., 2010; Hussman et al., 2011; Chen et al., 2014b). Here, we discuss the current understanding of how CAMs that belong to the cadherin-, the neurexin/neuroligin- and the immunoglobulin-superfamily regulate neuronal stability.

### Cadherin Superfamily Members Are Prominent Hits in Autism Risk Gene Lists

The genetic association of cadherins with autism strongly supports their central roles in the development of the nervous system including synaptogenesis, dendrite arborization and dendritic spine regulation (Arikkath and Reichardt, 2008; Suzuki and Takeichi, 2008; Basu et al., 2015; Friedman et al., 2015a; Seong et al., 2015). The cadherin superfamily is comprised of more than a hundred different genes, subdivided into several classes including classical cadherins, protocadherins and atypical cadherins (Hulpiau and van Roy, 2009; Hirano and Takeichi, 2012). Several copy number variations (CNVs) and single nucleotide polymorphisms (SNPs) are found in classical cadherins: *CDH2. CDH5. CDH8. CDH9, CDH10, CDH11*, and *CDH13* (Wang et al., 2010; Chapman et al., 2011; Hussman et al., 2011; Pagnamenta et al., 2011; Sanders et al., 2011; O’Roak et al., 2012b; Prandini et al., 2012; Connolly et al., 2013; Walker and Scherer, 2013; Crepel et al., 2014; Krumm et al., 2015); non-clustered protocadherins: *PCDH9, PCDH10*, and *PCDH19* (Morrow et al., 2008; Depienne et al., 2009; Camacho et al., 2012; O’Roak et al., 2012b; Prasad et al., 2012; Girirajan et al., 2013; van Harssel et al., 2013)*;* and an atypical cadherin, *FAT1* (Hussman et al., 2011; Neale et al., 2012; Cukier et al., 2014; Kenny et al., 2014). In general, the extracellular domain of cadherins contains five cadherin repeats/EC motifs that mediate Ca^2+^-dependent homophilic adhesion (Tanihara et al., 1994). However, heterophilic interactions between different sub-classes of classical cadherins create more combinations of interaction and function (Shimoyama et al., 2000). The cytosolic tail binds to catenins leading to the anchoring of the cadherin-catenin complex to the cytoskeleton (via β-catenin and α-catenin) and the clustering of cadherins in the plasma membrane (via p120-catenin) (Yap et al., 1998; Nelson, 2008; McCrea and Gu, 2010). The cadherin superfamily contains numerous cadherin members which impact neuronal structure and function from early neurite extension to the maintenance of mature synaptic networks (Basu et al., 2015; Friedman et al., 2015a). However, the pathways underlying disrupted cadherin signaling still requires further investigation. Here, we focus our discussion on the autism-associated cadherins shown to regulate neurite outgrowth and synapse morphogenesis.

*N*-cadherin, also known as cadherin 2 (CDH2), is the best studied classical cadherin. *N*-cadherin functions throughout the development of the nervous system, including neurite outgrowth, axon guidance, synaptogenesis and synaptic plasticity (Takeichi and Abe, 2005; Arikkath and Reichardt, 2008; Hirano and Takeichi, 2012; Friedman et al., 2015a). *N*-cadherin promotes dendritic outgrowth during development and is also required for activity-dependent dendrite expansion (Esch et al., 2000; Tan et al., 2010). *N*-cadherin is also required for the establishment of initial contacts between axons and filopodia followed by clustering at contact points to stabilize early synapses (Benson and Tanaka, 1998; Huntley and Benson, 1999; Togashi et al., 2002). Blocking *N*-cadherin adhesion in hippocampal neurons perturbs synapse formation and abolishes long-term potentiation (LTP)-induced stabilization of dendritic spines (Togashi et al., 2002; Mendez et al., 2010). Neural activity increases *N*-cadherin protein levels and dimerization leading to increased synapse number (Bozdagi et al., 2000). In mature synapses, *N*-cadherin is required for the persistence of dendritic spine enlargement and LTP (Bozdagi et al., 2000, 2010). Together with *N*-cadherin, CDH8 regulates the development of the hippocampal mossy fiber pathway (Bekirov et al., 2008). CDH8 also mediates assembly and maturation of corticostriatal synapses (Bekirov et al., 2008; Friedman et al., 2015b), whereas CDH9-mediated adhesion is involved in the formation and differentiation of dentate gyrus synapses on CA3 cells where it regulates synapse density, presynaptic bouton complexity and postsynaptic morphology (Williams et al., 2011). In contrast to CDH8 and CDH9, an RNAi screen for molecules required for synapse development identified CDH11 and CDH13 as positive regulators of glutamatergic synapse development (Paradis et al., 2007). Interestingly, *Cdh11*-deficient mice revealed enhanced LTP in the CA1 region of the hippocampus and mice show reduced fear- or anxiety-related behavior suggesting that CDH11 might restrict synaptic plasticity and efficacy (Manabe et al., 2000).

Protocadherins are the largest subgroup within the cadherin superfamily and are further subtyped into clustered (α-, β- and γ-PCDH) and non-clustered protocadherins (δ1- and δ2-PCDH) (Frank and Kemler, 2002). They share a similar structure to classical cadherins, but with six to seven cadherin domains/EC motifs. However, the cytosolic tails of protocadherins and cadherins do not show significant homology suggesting that they likely engage distinct intracellular signaling pathways. Protocadherins are highly expressed in the nervous system and localize to synapses. Based on their spatial and temporal expression pattern in the brain and on recent reports, protocadherins have roles in dendritic development and synaptic connections (Hirano et al., 1999; Frank and Kemler, 2002; Kim et al., 2007, 2011b; Keeler et al., 2015). For example, PCDH10 expression is regulated by neuronal activity and its function is crucial for forebrain axon outgrowth and the proper patterning of thalamocorticial projections (Uemura et al., 2007; Morrow et al., 2008). In addition, PCDH10 mediates synapse elimination by promoting proteasomal degradation of PSD-95 (Tsai et al., 2012a).

FAT atypical cadherin 1 (FAT1) belongs to the atypical cadherin family and consists of a huge extracellular domain comprising 34 cadherin domains/EC motifs (Tanoue and Takeichi, 2005; Sadeqzadeh et al., 2014). FAT1 expression is enriched during embryonic neurodevelopment and severe nervous system defects are found in FAT1-deficient mice (Ciani et al., 2003; Sadeqzadeh et al., 2014). At the cellular level, FAT1 localizes to cell–cell contacts as well as to the leading edge of lamellipodia and tips of filopodia to regulate cell polarity, cell migration, and cell-cell adhesion (Moeller et al., 2004; Tanoue and Takeichi, 2004). These functions are likely mediated through intracellular signaling via Ena/VASP proteins to regulate actin assembly and dynamics (Moeller et al., 2004; Tanoue and Takeichi, 2004). Other intracellular binding partners of FAT1 include the classical cadherin binding partner β-catenin as well as the synaptic scaffolding molecules Homer-1 and 3 (Hou et al., 2006; Schreiner et al., 2006).

### The Neurexin-Neuroligin Complex Is One of the Most Studied *Trans*-synaptic Adhesion Pairs in Autism

Neurexins are encoded by three genes (*NRXN1-3*) while the neuroligin family consists of four isoforms in mice (*Nlgn1-4*) and five isoforms in humans (*NLGN1-4X and 4Y*) (Südhof, 2008). Neurexins localize to presynaptic terminals and form heterophilic interactions with neuroligins, which are localized to the postsynaptic compartment (Ichtchenko et al., 1995, 1996; Nguyen and Sudhof, 1997). Presynaptic neurexins link synaptic adhesion with the synaptic vesicle release machinery via binding to PDZ-domain containing proteins (Hata et al., 1996; Butz et al., 1998; Dean et al., 2003; Missler et al., 2003). At the postsynaptic site, the neurexin-neuroligin complex induces clustering of scaffolding proteins, such as PSD-95, and recruits NMDA- and AMPA-receptors (Irie et al., 1997; Chih et al., 2005; Nam and Chen, 2005; Heine et al., 2008; Barrow et al., 2009; Mondin et al., 2011). Distinct neurexin-neuroligin complexes play discrete roles during synaptogenesis with neuroligin-1 regulating excitatory synapse formation and maturation, while neuroligin-2 and 3 mediate inhibitory synapse formation (Song et al., 1999; Scheiffele et al., 2000; Prange et al., 2004; Varoqueaux et al., 2004, 2006; Chih et al., 2005; Levinson et al., 2005; Barrow et al., 2009; Kwon et al., 2012; Gokce and Sudhof, 2013). Intriguingly, neurexin-1 can also bind to leucine-rich repeat transmembrane protein 2 (LRRTM2) and promote synapse formation (de Wit et al., 2009; Ko et al., 2009; Gokce and Sudhof, 2013). In addition to their synaptic roles, interaction of neurexin-1 and neuroligin-1 regulates neurite outgrowth (Gjorlund et al., 2012). Several mutations and CNVs in *NRXN1-3* have been found to be associated with ASD with the prevalence highest for mutations in *NRXN1* (Feng et al., 2006; Autism Genome Project Consortium et al., 2007; Kim et al., 2008; Yan et al., 2008; Ching et al., 2010; Pinto et al., 2010; Wisniowiecka-Kowalnik et al., 2010; Gauthier et al., 2011; Voineskos et al., 2011; Camacho-Garcia et al., 2012; Duong et al., 2012; Iossifov et al., 2012; Kong et al., 2012; Liu et al., 2012; Prasad et al., 2012; Schaaf et al., 2012; Vaags et al., 2012; Bena et al., 2013; Dabell et al., 2013; Girirajan et al., 2013; Jiang et al., 2013b; Koshimizu et al., 2013; Walker and Scherer, 2013; Cukier et al., 2014; De Rubeis et al., 2014; Egger et al., 2014; Imitola et al., 2014; Vinas-Jornet et al., 2014; Tammimies et al., 2015). Similarly, *NLGN1-4* genes have been implicated in the pathogenesis of ASD with *NLGN3* and *4* being the most prevalent (Jamain et al., 2003; Laumonnier et al., 2004; Ylisaukko-oja et al., 2005; Lawson-Yuen et al., 2008; Glessner et al., 2009; Yu et al., 2011, 2013; Leblond et al., 2012; O’Roak et al., 2012b; Steinberg et al., 2012; Yanagi et al., 2012; Girirajan et al., 2013; Jiang et al., 2013b; Iossifov et al., 2014; Kenny et al., 2014; Li et al., 2014a; Krumm et al., 2015; Sanders et al., 2015; Yuen et al., 2015). However, several reports also indicate the negative association of *NLGN3* and *4* with autism (Vincent et al., 2004; Gauthier et al., 2005; Blasi et al., 2006; Wermter et al., 2008; Avdjieva-Tzavella et al., 2012; Liu et al., 2013b; Xu et al., 2014). Further investigation is required to clarify this controversy.

Mutations in another member of the neurexin superfamily, contactin-associated protein-like 2 (*CNTNAP2/CASPR2*), have also been identified in individuals with autism (Alarcón et al., 2008; Arking et al., 2008; Bakkaloglu et al., 2008; Vernes et al., 2008; Li et al., 2010; Petrin et al., 2010; Poot et al., 2010; Nord et al., 2011; O’Roak et al., 2011; Peñagarikano et al., 2011; Whitehouse et al., 2011; Anney et al., 2012; Prasad et al., 2012; Girirajan et al., 2013; Sampath et al., 2013; Egger et al., 2014; Poot, 2014; Chiocchetti et al., 2015). CNTNAP2 is required for dendrite arborization and dendritic spine development and maintenance (Anderson et al., 2012; Gdalyahu et al., 2015). Mice deficient for *Cntnap2* show defects in spine stabilization and synaptic function resulting in several core ASD-like behaviors such as deficits in communication and social interaction, as well as repetitive behaviors (Peñagarikano et al., 2011; Gdalyahu et al., 2015; Varea et al., 2015).

### Immunoglobulin Superfamily of Cell Adhesion Molecules Participate Largely in Neuronal Circuit Formation

The immunoglobulin superfamily of CAMs (IgSF-CAMs), including contactin, L1CAM, NCAM or SynCAM, make up a third large group of *trans*-synaptic CAMs. IgSF-CAMs have been implicated in various processes during neural circuit formation, from neurite outgrowth and axonal navigation to synapse formation and plasticity (Rougon and Hobert, 2003).

The contactin (CNTN) subfamily consists of six members (CNTN1-6), each of which contain six Ig-like and four fibronectin III-like domains that are linked to the cell membrane via a glycosylphosphatidylinositol (GPI)-anchoring domain (Shimoda and Watanabe, 2009). While CNTN1 and 2 have been extensively studied in the context of neurite outgrowth, fasciculation, and axon guidance, less is known about the function of CNTN3-6 (Karagogeos, 2003; Shimoda and Watanabe, 2009; Mohebiany et al., 2014). However, *CNTN3-6* have been implicated as risk genes in ASD (Fernandez et al., 2004; Christian et al., 2008; Morrow et al., 2008; Glessner et al., 2009; Roohi et al., 2009; Cottrell et al., 2011; Hussman et al., 2011; van Daalen et al., 2011; Leblond et al., 2012; Prasad et al., 2012; Vaags et al., 2012; Cukier et al., 2014; Kashevarova et al., 2014; Nava et al., 2014; Poot, 2014; Hu et al., 2015; Liu et al., 2015a). CNTN4 is strongly expressed in a subset of olfactory sensory neurons where it guides proper targeting of axon terminals to the corresponding glomeruli for the formation of olfactory circuits (Kaneko-Goto et al., 2008). The *Cntn5* knockout mice display reduced fiber density and glutamatergic synapses in the auditory brainstem (Li et al., 2003; Toyoshima et al., 2009). CNTN6 is highly expressed in the postnatal cerebellum and plays an important role in the formation of synapses between parallel fibers and Purkinje cells (Takeda et al., 2003; Sakurai et al., 2009). Similarly, CNTN6 regulates the formation of glutamatergic synapses in the hippocampus and the orientation of apical dendrites of layer V pyramidal neurons in the visual cortex (Ye et al., 2008; Sakurai et al., 2010).

Neural cell adhesion molecule 2 (NCAM2) belongs to the NCAM family and is a paralog of NCAM1. Similar to other members of the Ig-superfamily, NCAMs contain five Ig- and two FN3-domains in the extracellular region and are differentially spliced to produce both transmembrane and GPI-anchored variants (Winther et al., 2012). NCAM2 is predominantly expressed in the brain and required for the formation and maintenance of axonal and dendritic compartmentalization in the olfactory glomeruli (Walz et al., 2006; Borisovska et al., 2011). In addition, NCAM2 regulates filopodia formation and neurite branching of cortical neurons via a CaMKII-dependent signaling pathway (Sheng et al., 2015). SNP and chromosomal deletion including *NCAM2* has been reported in individuals with autism (Haldeman-Englert et al., 2010; Hussman et al., 2011; Petit et al., 2015).

Adhesion molecules are a huge group of proteins that display many similarities in molecular structure and in signaling property. Depending on the cellular localization, functions of adhesion molecules range from neurite outgrowth and synapse/spine formation, to neuronal plasticity, further highlighting their importance in regulating the structural stability of neurons. However, whether these molecules function to compensate each other or are developmentally regulated is still not clear. The interesting question is whether the temporal and spatial expression patterns of these autism-associated adhesion molecules correlate with the affected developmental time frame and affected brain regions in ASD.

## Surface Receptors Signal Through Intracellular Signaling Pathways to Regulate Neuronal Stability

Establishment of synaptic connections and modification of their strength and stability is intimately related to the receptor populations in the plasma membranes of pre- and postsynaptic cell compartments. Thus, several ASD risk genes code for cell surface receptor proteins including the ionotropic glutamate receptors (iGluR) and the receptor tyrosine kinases (RTK).

### Glutamate Receptors: GRIN2B and the GRIK Genes

Glutamate-mediated ionotropic signaling occurs via activation of the glutamate-gated ion channel family which is divided into three subfamilies; the α-amino-3-hydroxy-5-methyl-4-isoxazolepropionic acid receptors (AMPARs), the *N*-methyl-D-aspartate receptors (NMDARs) and the kainate-type receptors (KARs) (Collingridge et al., 2009). The main function of these receptors focuses on the regulation of synaptic activity and plasticity, which in turn affect the structural stability of neurons. In particular, the genetic association of the *GRIN2B* gene, which encodes the GluN2B subunit of NMDARs, and the *GRIK2/4* genes that encodes the GluK2 and 4 subunits of KARs with autism has been established (Jamain et al., 2002; Shuang et al., 2004; Holt et al., 2010; Myers et al., 2011; O’Roak et al., 2011, 2012a; Tarabeux et al., 2011; de Ligt et al., 2012; Griswold et al., 2012; Prasad et al., 2012; Talkowski et al., 2012; Yoo et al., 2012; Dimassi et al., 2013; De Rubeis et al., 2014; Kenny et al., 2014; Li et al., 2014a; Namjou et al., 2014; Poot, 2014; Aller et al., 2015; Pan et al., 2015).

NMDARs are composed of an obligatory GluN1 subunit and one or more GluN2 (GluN2A-GluN2D) subunits with the majority of the composition being GluN1/2A/2B (Buller et al., 1994; Petralia et al., 1994; Luo et al., 1997). The composition of GluN2 subunits are developmentally regulated and critically determine the synaptic properties (Laurie and Seeburg, 1994; Sheng et al., 1994; Li et al., 1998). The GluN2B subunit expresses early during development gradually being replaced by GluN2A indicating its role in the formation of neuronal circuitry (Sheng et al., 1994; Li et al., 1998; Bustos et al., 2014). Overexpression or knockdown of GluN2B alters dendrite arborization in neurons both *in vivo* and *in vitro* (Ewald et al., 2008; Espinosa et al., 2009; Sepulveda et al., 2010; Bustos et al., 2014). GluN2B is also required for the formation of dendritic spines, maturation of synapses, and the proper molecular compositions of several postsynaptic proteins (Akashi et al., 2009; Espinosa et al., 2009; Brigman et al., 2010; Kelsch et al., 2012). In turn, GluN2B is crucial for maintaining proper synaptic plasticity (Brigman et al., 2010; Ohno et al., 2010; Wang et al., 2011a; Yang et al., 2012a; Ryan et al., 2013; Dupuis et al., 2014). *GRIN2B*, an autism-risk gene, further suggests that pathways involved in early circuitry formation may be vulnerable targets in autism. Selective inhibition of GluN2B function has been shown to restore dendritic spine loss and associated behavior alterations in several experimental conditions providing insights to the potential therapeutic targets to correct some ASD phenotypes (Chen et al., 2011; Iafrati et al., 2014; Gupta et al., 2015).

Kainate-type receptors regulate axonal filopodia motility of hippocampal mossy fibers in response to neuronal stimulation during synaptogenesis (Tashiro et al., 2003). KAR subunits, in particular GluK2, interacts with structural elements of the synapse; such as the PSD-95 and SAP-102 scaffolding molecules, as well as the *N*-cadherin and β-catenin adhesion molecules (Carta et al., 2014; Pahl et al., 2014), indicating that *Grik* genes are involved in processes that regulate synapse architecture and stability. Indeed, GluK2 regulates hippocampal synapse maturation and stability (Huettner, 2003; Contractor et al., 2011; Lanore et al., 2012; Lerma and Marques, 2013). Animals with deficient GluK2 proteins exhibit a delay in the postnatal maturation of synaptic contacts between MF-CA3 in the hippocampus, suggesting that the expression of the GluK2 is important for the establishment of normal morphology and function of synaptic networks in the hippocampus (Contractor et al., 2001; Lanore et al., 2012). Expression of the GluK4 is mainly restricted to mossy fiber synapses in the hippocampal CA3 region where it co-assembles with GluK2 in functional pre- and postsynaptic GluK2/4 receptor complexes (Darstein et al., 2003). Mice with forebrain GluK4 overexpression exhibit altered synaptic transmission and display several autistic-like behaviors including social impairment, enhanced anxiety, and depressive states, coinciding with the finding of *GRIK4* duplications in individuals with ASD (Griswold et al., 2012; Aller et al., 2015). Even though the phenotypes resulting from *Grik* gene dysfunction in mice are in the same general categories with symptoms of ASD, further investigation about the molecular consequences of impairments in GluK proteins in ASD is required for developing future therapeutic interventions.

### Receptor Tyrosine Kinases: The NTRK Genes

Tyrosine receptor kinases (Trks) mediate neurotrophic growth factor-induced signaling via dimerization and trans-autophosphorylation of Tyr residues on the intracellular domains of the receptor and subsequent activation of intracellular signaling pathways (Deinhardt and Chao, 2014). This results in a number of neurogenic events, such as synaptic plasticity, maturation and stability, dendritic and axonal growth and differentiation as well as cell survival and maintenance (Martinez et al., 1998; Deinhardt and Chao, 2014). The Trk family consists of three proteins; TrkA, B and C, which are expressed by the neurotrophic tyrosine receptor kinase genes (*NTRK. 1. 2* and *3*, respectively. Each Trk receptor interacts selectively with a different neurotrophin resulting in preferential interaction pairs: TrkA is activated by NGF, TrkB by BDNF, and TrkC by NT-3 (Deinhardt and Chao, 2014). Considering its well-documented function in synaptophysiology (Minichiello, 2009), it would be reasonable to suspect a correlation between genetic variations in *NTRK2* and ASD. However, to date, only one study has reported a weak association between *NTRK2* mutations and ASD (Correia et al., 2010), while other studies were unable to confirm that link (Chakrabarti et al., 2009). Alternatively, a growing body of evidence generated from genetic evaluation of ASD risk genes has identified *NTRK3*, the gene coding for TrkC, as a plausible candidate in autism (Chakrabarti et al., 2009; Hussman et al., 2011; Vardarajan et al., 2013).

In the mammalian brain, TrkC (as well as other neurotrophic receptors) is present both as full length catalytically active receptor, as well as a splice variant that lacks the Tyr kinase domain and is catalytically inactive (Ichinose and Snider, 2000). Interestingly, knockout of the non-catalytic TrkC isoform in mice yields a more severe phenotype than does the depletion of the kinase-active receptor, indicating that TrkC has important functions beyond the ability to convey classical RTK signaling (Faux et al., 2007; Deinhardt and Chao, 2014). Indeed, recent studies have begun to elucidate the function assigned to non-catalytic isoforms by demonstrating a role for TrkC in synaptic adhesion complexes (Takahashi and Craig, 2013). Postsynaptic TrkC interacts across the presynaptic cleft with protein tyrosine phosphatase (PTP) σ to form an adhesion complex crucial for development and stability of excitatory, but not inhibitory, synapses (Takahashi et al., 2011; Coles et al., 2014). Formation of this adhesion complex is enhanced by the presence of the TrkC ligand, NT-3, which facilitates glutamatergic presynaptic assembly and function (Ammendrup-Johnsen et al., 2015). NT-3 binding to kinase domain-truncated TrkC isoforms has also been shown to induce cytoskeletal changes via recruitment of the scaffold protein tamalin, leading to activation of Arf6 and induction of Rac1-GTP (Esteban et al., 2006). Interestingly, the expression of non-catalytic TrkC relative to the kinase active isoform is upregulated during the second and third postnatal weeks, the most intense period of synaptogenesis, indicating that expression of the different *Ntrk3* gene products is temporally associated with synapse formation (Valenzuela et al., 1993; Menn et al., 2000). Recent studies also found that NT-3-TrkC signaling between presynaptic granule neurons and postsynaptic Purkinje cells controls dendrite morphogenesis in cerebellum (Joo et al., 2014). Although no studies have evaluated the ratio of non-catalytic to catalytic TrkC receptors in individuals with ASD, it might be speculated that certain genetic variants could cause imbalances in the expression patterns of *NTRK3* isoforms.

Surface receptors respond to extracellular signals such as neurotransmitters and trophic factors to activate downstream signaling pathways to diversify the cellular responses. Each receptor may have a unique signaling pathway associated with it and therefore, the mutations on selective receptors provide us with clues about which signaling pathways may be more susceptible to perturbations in ASD. Thus, identifying the downstream effectors and signaling pathways that are affected by these autism-associated receptor mutants should be an important direction of future investigation.

## Signaling Molecules Actively Regulate Dendritic Spine and Dendrite Morphology

### Protein Kinases

The dual-specificity tyrosine-(*Y*)-phosphorylation-regulated kinase 1a (DYRK1A) is one of the isoforms in DYRK family and is a human homolog of the *Drosophila* kinase minibrain (MNB) (Shindoh et al., 1996). *DYRK1A* was first described as a cadidate gene for intellectual disability in Down syndrome because of its location on the “Down syndrome critical region” of chromosome 21 (van Bon et al., 1993; Shindoh et al., 1996; Hammerle et al., 2003). Interestingly, recent genetic analyses suggest that *DYRK1A* is also a risk gene in ASD (Iossifov et al., 2012; O’Roak et al., 2012a,b; Chen et al., 2014a; Krumm et al., 2014; Redin et al., 2014; Bronicki et al., 2015; van Bon et al., 2016). Expression of *Dyrk1a* in mouse brain is limited to early developmental periods and can promote neurite formation (Okui et al., 1999; Hammerle et al., 2003; Gockler et al., 2009). In addition, DYRK1A phosphorylates N-WASP, a cytoskeletal protein, to inhibit spine formation in primary hippocampal neurons (Park et al., 2012). Pyramidal neurons in *Dyrk1a^+/-^* mouse cortex have reduced dendritic branches and dendritic spine density, which potentially causes the reduced brain size in these mice (Fotaki et al., 2002; Benavides-Piccione et al., 2005). On the other hand, overexpression of DYRK1A in mice causes increased spine density in cortical pyramidal neurons, and these animals show prefrontal deficits including significant impairment of spatial learning and cognitive flexbitiliy (Altafaj et al., 2001; Thomazeau et al., 2014). However, overexpressing DYRK1A in primary cortical mouse neurons significantly reduces dendrite complexity through disruption of REST/NRSF levels and REST/NRSF-SWI/SNF chromatin remodeling complex (Lepagnol-Bestel et al., 2009).

The Cyclin-dependent kinase-like 5 (CDKL5) is a serine/threonine kinase, also known as serine/threonine kinase 9 (STK9). Mutations of *CDKL5* have been associated with several X-linked neurodevelopmental disorders, as well as ASD (Weaving et al., 2004; Scala et al., 2005; Archer et al., 2006; Russo et al., 2009; Sprovieri et al., 2009; Schaaf et al., 2011; Bahi-Buisson and Bienvenu, 2012; Bartnik et al., 2012; Maortua et al., 2012; Carvill et al., 2013; Epi et al., 2013; Piton et al., 2013; Zhao et al., 2014; Codina-Sola et al., 2015; Szafranski et al., 2015). Expression of CDKL5 is enriched in the brain and increases gradually following development (Lin et al., 2005; Rusconi et al., 2008). In addition to a catalytic domain, CDKL5 contains nuclear localization and export signals and can shuttle between the cytoplasm and nucleus. In the nucleus, CDKL5 phosphorylates methyl-CpG-binding protein 2 (MECP2), a causative gene for Rett syndrome (see below), providing a suggestive molecular mechanism associated with the condition (Mari et al., 2005). In the cytosol, CDKL5 postively regulates neurite outgrowth and dendritic arborization via binding with Rac1 (Chen et al., 2010b). With this broad influence on neuronal function, *Cdkl5* null mice have several defects ranging from neuronal survival, dendritie maturation, spine stability, synaptic plasticity, and behaviors (Amendola et al., 2014; Fuchs et al., 2014; Della Sala et al., 2016). Treatment of *Cdkl5* null mice with insulin-like growth factor 1 (IGF-1) or the glycogen synthase kinase 3β (GSK3β) inhibitor can rescue these defective phenotypes (Della Sala et al., 2016; Fuchs et al., 2015). Furthermore, CDKL5 has been shown to bind to palmitoylated-PSD-95 and this interaction is important for synaptic targeting of CDKL5 and spine formation (Zhu et al., 2013).

### Phosphatase: PTEN

Phosphatase and tensin homolog (PTEN) is a dual-specificity lipid/protein tyrosine phosphatase that negatively regulates the phosphatidylinositol 3-kinase (PI3K)/AKT/mammalian target of the rapamycin (mTOR) pathway to control cellular function (Maehama and Dixon, 1998, 1999; Stambolic et al., 1998; Vazquez and Sellers, 2000; Downes et al., 2001; Leslie and Downes, 2002; Hoeffer and Klann, 2010). PTEN was first identified as a tumor suppressor (Li et al., 1997) but later also found to be associated with neurodevelopmental conditions such as epilepsy, macrocephaly, and autism (Goffin et al., 2001; Butler et al., 2005; Buxbaum et al., 2007; Orrico et al., 2009; Varga et al., 2009; McBride et al., 2010; Redfern et al., 2010; Stein et al., 2010; Schaaf et al., 2011; O’Roak et al., 2012b; Busa et al., 2013; De Rubeis et al., 2014; Hobert et al., 2014; Marchese et al., 2014; Vanderver et al., 2014; Codina-Sola et al., 2015; D’Gama et al., 2015; Johnston and Raines, 2015; Krumm et al., 2015; Spinelli et al., 2015; Tammimies et al., 2015; Cupolillo et al., 2016; Schwerd et al., 2016; Tilot et al., 2016). PTEN expression in the brain is positively correlated with the developmental stages of neuronal dendrite formation and synaptogenesis suggesting a role in regulating neuronal function (Perandones et al., 2004). PTEN plays a critical role in regulating the stability of dendritic spines and synaptic activity. PTEN overexpression in hippocampal CA1 pyramidal neurons results in a decrease in spine density (Zhang et al., 2012). Deleting PTEN in cortical and hippocampal neurons causes loss of neuronal polarity and general neuronal hypertrophy, including increases in dendrite arborization and spine density (Jaworski et al., 2005; Kwon et al., 2006; Fraser et al., 2008; Zhou et al., 2009). Selective deletion of PTEN in dentate granule neurons results in increased spine density and synaptic activity, as well as increased mossy fiber sprouting (Luikart et al., 2011; Pun et al., 2012). Knockdown of PTEN in basaolateral amygdala and dentate gyrus, however, does not affect spine density, but spine morphology is dramatically altered with an increase of mature mushroom-shaped spines (Haws et al., 2014). The *Pten* knockout in cerebellum also results in significant alterations in neuronal morphology of Purkinje cells including swelling of dendrites, and an increase of axonal bouton and dendritic spine size (Cupolillo et al., 2016). The impact of PTEN on spine stability is dependent on the phosphorylation status of its serine/threonin residues and the PDZ-binding motif in its C-terminus (Zhang et al., 2012). The phenotypes observed following PTEN deletion result from hyperactivation of the PI3K/AKT/mTOR pathway and inhibition of this molecular pathway is sufficient to rescue these phenotypes (Jaworski et al., 2005; Kwon et al., 2006; Zhou et al., 2009; Pun et al., 2012).

The identification of the vulnerable intracellular signaling pathways will aid us in the pursuit to find new therapeutic drug targets in patients with ASD. Interestingly, a variety of gene mutations result in disruption of the mechanistic pathways that these signaling molecules participate in. Therefore, several of the experimental pharmacological agents currently proposed as possible treatment strategies for autistic phenotypes are targeting these signaling molecules (see “Perspectives”).

## Synaptic Proteins Regulate Synaptic Function to Maintain Neuronal Stability

### Scaffolding Proteins Provide Supporting Roles to Connect Structural and Signaling Molecules

Synaptic signaling processes are key to proper neural function. Some pivotal components of synapses are postsynaptic scaffolding proteins, which cluster neurotransmitter receptors, cell adhesion proteins, ion channels and cytoskeletal molecules to a confined postsynaptic region (Kim and Sheng, 2004; Sheng and Hoogenraad, 2007). Dysfunction in scaffolding proteins often has a huge impact on neuronal function, including neuronal morphology and synaptic plasticity (Ting et al., 2012). Emerging evidence has recently linked ASD with mutations of several genes encoding scaffolding proteins as described below.

*SHANK* genes encode three large scaffolding proteins, SHANK1-3, that contain ankyrin repeats, the SH3 domain, the PDZ domain, the proline-rich domain, and the SAM domain (Naisbitt et al., 1999; Sheng and Kim, 2000; Baron et al., 2006). These multiple putative protein interaction domains enable shank proteins to function as a bridge linking inotropic glutamate receptors, PSD-95, SAPAPS (Naisbitt et al., 1999), Homers (Tu et al., 1999; Hayashi et al., 2009) and the cytoskeleton (Böckers et al., 2001; Qualmann et al., 2004). Altered function of all three *SHANK* genes have been implicated in autism, with *SHANK3* showing the highest prevalence (Moessner et al., 2007; Gauthier et al., 2009; Awadalla et al., 2010; Berkel et al., 2010, 2012; Pinto et al., 2010; Schaaf et al., 2011; Waga et al., 2011; Leblond et al., 2012, 2014; Prasad et al., 2012; Sanders et al., 2012; Sato et al., 2012; Boccuto et al., 2013; Koshimizu et al., 2013; Coe et al., 2014; De Rubeis et al., 2014; Guilmatre et al., 2014; Li et al., 2014a; Cochoy et al., 2015; Krumm et al., 2015; Nemirovsky et al., 2015; Yuen et al., 2015). In general, SHANK2 and 3 promote dendritic spine formation, whereas SHANK1 promotes dendritic spine head size enlargement (Sala et al., 2001; Roussignol et al., 2005; Hung et al., 2008; Verpelli et al., 2011). SHANK3 also binds to a synaptic scaffold, Densin-180, to inhibit the Densin-180-induced dendrite arborization (Quitsch et al., 2005). Disruption of the *SHANK3* gene is associated with the 22q13.3 deletion syndrome, characterized by severe expressive-language delay and mild cognitive challenges (Bonaglia et al., 2001). Several genetically manipulated *Shank3* mutant mouse models were developed to study the role of *SHANK3* mutations in ASD (Bangash et al., 2011; Peça et al., 2011; Wang et al., 2011b; Jiang and Ehlers, 2013; Peixoto et al., 2016). Neurons in these mice have morphological alterations in dendritic spines resulting in LTP deficiency and defects at striatal synapses. Furthermore, these animals display several behavioral deficits including abnormal vocalization, dyadic social interaction, and compulsive-repetitive behaviors. Intriguingly, re-expressing *Shank3* in adulthood is sufficient to restore parts of the autism-related phenotypes in mice (Mei et al., 2016). This study sheds light on the application of gene therapy for individuals with *SHANK3* mutations. Mechanistically, several actin regulators including Abp1, cortactin, cofilin, and Rac1, have altered expression or activity associated with *Shank3* deficiency or autism-related mutations that contribute to dendritic spine reduction and synaptic dystrophy (Haeckel et al., 2008; Durand et al., 2012; Duffney et al., 2015).

Disks large-associated protein 2 (DLGAP2), also known as synapse-associated protein 90/postsynaptic density-95-associated proteins (SAPAP2), is a postsynaptic adapter protein in mammalian brains (Kindler et al., 2004). DLGAP2 directly interacts with DLG4 (also known as PSD-95) and Shank proteins to form the Dlg4-Dlgap-Shanks complex important for maintaining the PSD structure (Kim et al., 1997; Takeuchi et al., 1997; Boeckers et al., 1999). The *Dlgap2^-/-^* mice have reduced spine density in the orbitofrontal cortex accompanied with downregulation of synaptic proteins, Homer1 and αCaMKII, as well as receptors, NR1 and GluR1, and exhibit exacerbated aggressive behaviors (Jiang-Xie et al., 2014). Molecular and genetic studies have demonstrated that alterations in *DLGAP2* are involved the pathophysiology of various psychiatric conditions, including schizophrenia, Alzheimer’s disease, post-tramautic syndrome, and pediatric obsessive-compulsive disorder (Chertkow-Deutsher et al., 2010; Wu et al., 2013; Li et al., 2014b; Chaudhry et al., 2015). Rare *de novo* CNVs, deletions, and duplications of *DLGAP2* have been reported in individuals with ASD, but how mutations of *DLGAP2* contribute to autism is still largely unknown (Marshall et al., 2008; Ozgen et al., 2009; Chien et al., 2010; Pinto et al., 2010; Cukier et al., 2014).

### Molecules Regulating Synaptic Vesicles Are Implicated in the Regulation of Neurite Outgrowth

Neurotransmitter release is regulated by the cycling of synaptic vesicles at the axonal terminal. The regulation of synaptic vesicles contains several steps and requires precise interaction of several specialized proteins, including SNARE complex for membrane fusion and syntaxin for vesicle docking. *STXBP5* encodes a syntaxin-binding protein, tomosyn that negatively regulates neurotransmitter release by forming a syntaxin-SNAP25-tomosyn complex (Fujita et al., 1998; Sakisaka et al., 2004; Yizhar et al., 2004; Yamamoto et al., 2009, 2010; Bielopolski et al., 2014). Neuron-specific tomosyn deletion in mouse hippocampal dentate gyrus impairs spatial learning and memory, whereas tomosyn knockdown in dentate gyrus decreases synaptic plasticity of mossy fibers (Barak et al., 2013; Ben-Simon et al., 2015). Tomosyn also regulates SNARE complexes via ROCK phosphorylation of syntaxin-1 to control neurite outgrowth (Sakisaka et al., 2004). Recent genetic studies have identified the association of *STXBP5* and ASD (Davis et al., 2009; Cukier et al., 2014; De Rubeis et al., 2014).

*PRICKLE1* encodes PRICKLE1 protein, which has been traditionally thought to regulate the Wnt/beta-catenin signaling pathway to control epithelial planar cell polarity and cell migration during neural tube formation (Heitzler et al., 1993; Carreira-Barbosa et al., 2003; Veeman et al., 2003; Jenny et al., 2005). Intriguingly, the *Prickle1^+/-^* mice exhibit autism-like behaviors, which may result from disrupted interaction with synapsin, a regulator of neurotransmitter release, suggesting that PRICKLE1 plays a critical role in synaptic vesicle regulation (Paemka et al., 2013). In addition, knockdown of PRICKLE1 in mice results in reduced axonal and dendrite formation in hippocampal neurons (Liu et al., 2013a). More recently, variants of *PRICKLE1* have been found in individuals with autism (Cukier et al., 2014; Toma et al., 2014).

Synaptic scaffolds are crucial not only to maintain the structural stability of dendritic spines and synapses but also to link the signaling molecules and receptors to efficiently act in response to certain extracellular stimuli. Mutations in these molecules may disrupt several different signaling pathways and result in wide range of cellular defects, which sometimes are not limited to ASD. In addition, couple autism-associated genes that have been shown to regulate synaptic vesicles also play roles in neurite outgrowth or synaptic plasticity thereby regulating the structural stability of neurons. An interesting direction of investigation is whether the regulation of synaptic vesicles represents one of the key vulnerable cellular pathway that contributes to the alteration of neuronal structures in ASD.

## Specific Syndromic Disorder Related Genes

Several autism-related neurodevelopmental disorders, such as Fragile X, Rett, Angelman syndromes (AS), and tuberous sclerosis are caused by a highly penetrable mutation of a single gene, e.g., *FMR1* in Fragile X (Verkerk et al., 1991; Gedeon et al., 1992), *MECP2* in Rett (Amir et al., 1999), *UBE3A* in AS (Kishino et al., 1997), and *TSC1/2* in tuberous sclerosis complex (TSC; Povey et al., 1994). In recent DMS-5 criteria, however, ASD condition has been separated out from these single gene related disorders. Interestingly, these molecules all have a major function in regulating gene expression or protein synthesis, which in turn widely affects the structural stability of neurons. Because of the comorbidity between ASD and these single gene related disorders, we also review the current understanding of these genes and discuss how alterations of these genes may impair the structural integrity of neurons.

### FMRP

FMRP is a RNA-binding protein encoded by *FMR1* gene (Ashley et al., 1993; Siomi et al., 1993). Mutations of the *FMR1* gene in humans result in CGG repeat polymorphisms or in the deletion of FMRP protein contributing to Fragile X syndrome, the most common inherited form of intellectual disability (Verkerk et al., 1991; Gedeon et al., 1992). Interestingly, people with Fragile X syndrome often exhibit autistic behaviors and mutations of *FMR1* genes are also found in several cases of ASD (Vincent et al., 1996; Feinstein and Reiss, 1998; Rogers et al., 2001; Hatton et al., 2006; Chaste et al., 2012; Yu et al., 2013). The absence of FMRP is associated with widespread morphological changes of dendrites and dendritic spines in different brain regions, including cortex (Comery et al., 1997; Weiler and Greenough, 1999; Irwin et al., 2000; Greenough et al., 2001; Nimchinsky et al., 2001; Galvez et al., 2003; Galvez and Greenough, 2005; McKinney et al., 2005; Bureau et al., 2008; Cruz-Martin et al., 2010; Pan et al., 2010; Qin et al., 2011; Berman et al., 2012; Amiri et al., 2014; Wijetunge et al., 2014), hippocampus (Braun and Segal, 2000; Segal et al., 2003; Antar et al., 2006; Grossman et al., 2010; Levenga et al., 2011a; Amiri et al., 2014), and cerebellum (Koekkoek et al., 2005).

Upon mGluR-activation, the local translation of *Fmr1* in dendrite and dendritic spines is crucial for maintaining dendritic structure (Antar et al., 2004; Bear et al., 2004; Tucker et al., 2006; Osterweil et al., 2010; Pop et al., 2014). The hyperactivation of mGluR5 signaling, as well as the neuronal and behavioral deficits resulting from FMRP deficiency can be corrected by application of a mGluR5 antagonists (Bear et al., 2004; Bear, 2005; Tucker et al., 2006; Dolen et al., 2007; Price et al., 2007; Wilson and Cox, 2007; Auerbach and Bear, 2010; Osterweil et al., 2010; Levenga et al., 2011b; Michalon et al., 2012, 2014; Ronesi et al., 2012; Lohith et al., 2013; Pop et al., 2014). The activation of FMRP subsequently regulates the local synthesis of several other synaptic proteins, including AMPAR, CaMKII, and PSD95, which in turn modulates the activity-dependent dynamics and plasticity of dendritic spines (Muddashetty et al., 2007; Nakamoto et al., 2007; Kao et al., 2010; Ifrim et al., 2015). In addition, the Rac-PAK pathway is upregulated and coincides with the disruptive dendritic phenotype in the absence of FMRP (Lee et al., 2003; Hayashi et al., 2007; Chen et al., 2010a; Bongmba et al., 2011). A rare deletion and several variants of cytoplasmic FMR1 interacting protein 1 (*CYFIP1*) are also found in cases of ASD further suggesting genes involved in the FMRP signaling pathway are prevalent risk factors (van der Zwaag et al., 2010; Leblond et al., 2012; Toma et al., 2014; Waltes et al., 2014; Wang et al., 2015). Interestingly, CYFIP1 mediates FMRP-dependent protein translation to regulate the dendritic complexity and the stability of dendritic spines (Napoli et al., 2008; De Rubeis et al., 2013; Pathania et al., 2014). Overexpression of CYFIP1 results in an increase of dendritic branching and dendritic spine density (Oguro-Ando et al., 2015). Thus, the understanding of the molecular mechanisms regulated by FMRP provides insights into how mutations of *FMR1* in ASD may affect neuronal function and contribute to autistic behaviors.

### MECP2

Methyl-CpG binding protein 2 (MECP2) is a transcriptional factor that has multiple functions in gene regulation (Meehan et al., 1989; Nan et al., 1997, 1998; Young et al., 2005; Chahrour et al., 2008; Cheng et al., 2014). Mutations of *MECP2* gene contribute to 90% of cases with Rett syndrome, which is a severe developmental disorder exhibiting autistic phenotypes (Amir et al., 1999; Shahbazian and Zoghbi, 2001; Van den Veyver and Zoghbi, 2001). Interestingly, the *MECP2* duplication syndrome also exhibit phenotypes that resemble those with ASD (Ramocki et al., 2009; Peters et al., 2013; Lombardi et al., 2015). Mutations and CNVs of *MECP2*, including duplication of the gene, have been identified in people with ASD without the diagnoses of either Rett or *MECP2* duplication syndromes (Carney et al., 2003; Zappella et al., 2003; Shibayama et al., 2004; Swanberg et al., 2009; Campos et al., 2011; Schaaf et al., 2011; Cukier et al., 2012; Hanchard et al., 2012; Lim et al., 2013; Yu et al., 2013). Most studies on MECP2 function focus on the understanding of the etiology of Rett syndrome and the *MECP2* duplication syndrome. Upregulation or downregulation of MECP2 dramatically alters the dendritic and axonal architecture of neurons and significantly disrupts the connectivity of neuronal networks (Fukuda et al., 2005; Jugloff et al., 2005; Ballas et al., 2009; Belichenko et al., 2009; Chapleau et al., 2009; Kishi and Macklis, 2010; Cohen et al., 2011; Marshak et al., 2012; Nguyen et al., 2012; Stuss et al., 2012; Jiang et al., 2013a; Baj et al., 2014). Overexpression of BDNF appears to restore the dendritic defects in *Mecp2*-null condition suggesting a molecular mechanism regulated by MECP2 to maintain the structural stability of neurons (Zhou et al., 2006; Larimore et al., 2009; Gao et al., 2015). Synaptic plasticity is also regulated by the expression or the phosphorylation of MECP2 (Collins et al., 2004; Dani et al., 2005; Asaka et al., 2006; Moretti et al., 2006; Chao et al., 2007; Zhang et al., 2008; Li et al., 2011; Noutel et al., 2011; Blackman et al., 2012; Na et al., 2012, 2013; Qiu et al., 2012; Zhong et al., 2012; Della Sala and Pizzorusso, 2014; Deng et al., 2014; De Filippis et al., 2015). Whether a similar mechanism to Rett or *MECP2* duplication syndromes is altered in ASD individuals with *MECP2* mutations needs to be further evaluated.

### UBE3A

*UBE3A* gene is a paternally imprinted gene located at human chromosome 15 and encodes a member of the E3 ubiquitin ligase proteins (Huibregtse et al., 1993; Albrecht et al., 1997). Because *UBE3A* is selectively imprinted in mature neurons, epigenetic regulation of *UBE3A* has been associated with several neurodevelopmental disorders (Albrecht et al., 1997; LaSalle et al., 2015). Mutations resulting in loss-of-function in the maternally expressed copy of *UBE3A* causes AS, a severe developmental disorder characterized by delayed development, intellectual disability, severe speech impairment, and ataxia (Kishino et al., 1997). Maternal duplication of *UBE3A* results in Dup15q syndrome, a developmental disorder that has many similarities with AS but also exhibits several autistic traits (Cook et al., 1997; Wang et al., 2008; Hogart et al., 2010; Smith et al., 2011; Urraca et al., 2013; Al Ageeli et al., 2014; Germain et al., 2014). Coincidently, several genome-wide studies from individuals with autism identify *UBE3A* as an autism-risk gene (Nurmi et al., 2001; Glessner et al., 2009; Schaaf et al., 2011; Kelleher et al., 2012; Carvill et al., 2013; Iossifov et al., 2014; Yuen et al., 2015). In addition to its function of ubiquitin ligase to catalyze the protein degradation step, UBE3A also can act as a transcriptional coactivator for the nuclear hormone receptor superfamily of transcription factors (Nawaz et al., 1999). UBE3A localizes both in the nucleus and cytosol, including dendrite and pre- and post-synaptic compartments in neurons to regulate dendrite and dendritic spine morphology (Dindot et al., 2008; Valluy et al., 2015). Although maternal deletion of *Ube3a* does not affect dendrite arborization in mouse brains, knockdown of UBE3A in cultured neurons results in defects of dendrite polarization in pyramidal neurons (Dindot et al., 2008; Miao et al., 2013). Maternal-deficiency of *Ube3a* in mouse brain, however, shows defects in dendritic spine development in the cortex, hippocampus, and cerebellum (Dindot et al., 2008; Kim et al., 2016). Furthermore, several neuronal substrates for UBE3A have been identified, including Arc (Greer et al., 2010), the Rho-GEF Pbl/ECT2 (Reiter et al., 2006), Ephexin5 (Margolis et al., 2010), and TSC2 (Zheng et al., 2008). Their regulation by UBE3A provides molecular mechanisms to explain how synaptic integrity is maintained and how alteration of this interaction contributes in part to neuronal phenotypes in neurodevelopmental disorders. In addition, a recent study demonstrates that a PKA phosphorylation-defective mutation on *UBE3A* found in an individual with autism resulted in an increase of dendritic spine density (Yi et al., 2015).

### TSC1/TSC2

*TSC1* and *TSC2* genes encode protein harmartin and tuberin, respectively, and they bind and function together (European Chromosome 16 Tuberous Sclerosis Consortium, 1993; van Slegtenhorst et al., 1997; van Slegtenhorst et al., 1998). Mutations of *TSC1* or *TSC2* genes cause an autosomal dominant disorder TSC, which is characterized by hamartomas in various organs (Povey et al., 1994; Crino et al., 2006). Some patients with TSC exhibit several neurological problems including autism (Smalley, 1998; Bolton, 2004). Similarly, mutations of *TSC1* or *TSC2* are also found in several ASD cases (Smalley, 1998; Serajee et al., 2003; Schaaf et al., 2011; Kelleher et al., 2012; O’Roak et al., 2012b; Koshimizu et al., 2013; Brett et al., 2014; Toma et al., 2014; Krumm et al., 2015). TSC1/2 act upstream as to suppress the mammalian target of rapamycin (mTOR) pathway and mTOR inhibitors have been promising therapeutic agents to ameliorate some symptoms in TSC (Gao et al., 2002; Meikle et al., 2008; Curatolo et al., 2016). One of the mTOR inhibitors, rapamycin, has also been shown to correct the autistic-like synaptic pruning deficits in *Tsc2^+/-^* mice (Tang et al., 2014). In addition to the mTOR pathway being the key downstream target for TSC1/2, LIMK-cofilin pathway is altered upon deletion of TSC1 or TSC2 and enlargement of somas and dendritic spines are observed (Tavazoie et al., 2005). The *Tsc2* mutations result in a loss of Purkinje cells and *Tsc1* mutant mice have increased dendritic spine density, which correlates with cerebellar dysfunction and several autistic-like behaviors in animals (Tsai et al., 2012b; Reith et al., 2013). TSC1/2 also negatively regulates neurite and axonal outgrowth (Floricel et al., 2007; Choi et al., 2008). It has yet to be determined whether *TSC1/2* mutations in ASD correlate with perturbations of neuronal structures.

## Perspectives

Diagnosis of ASD cases has risen dramatically in recent years. The increased awareness of the symptoms and the broader definition of the spectrum may be major contributing factors for the rising number of ASD cases. Thus, there is an increased interest on understanding the etiologies of ASD. It is widely accepted that the genetic component plays a major role in ASD, however, except for the direct inheritance of some syndromic conditions, it is difficult to identify risk factors for autism. It is possible due to the low sample size and the high heterogeneity of genetic variances to have sufficient statistical power to make conclusive correlations (Geschwind and State, 2015). Among those autism-risk genes identified to date, some of the autism associations are due to *de novo* mutations, and some are familial variants (**Table 4**). Whether the inheritance pattern exhibits a risk factor is still not clear, however, the diverse gene mutations found in different individuals with autism suggest that instead of focusing on the genes *per se*, identifying the vulnerable pathways that these genes regulate may provide better clues toward understanding the contributing cellular and molecular changes that reserve in the autism phenotypes. The cellular defects resulting from different combinations of gene mutations contribute to the diverse phenotypes observed in autism. The heterogeneity of symptoms in ASD further complicates the diagnosis and treatment. However, understanding how autism-associated genes function in the regulation of key cellular pathways will provide insights to how therapeutic intervention can be more targeted and efficient to treat affected individuals.

**Table 4:** Inheritance pattern of autism-risk genes that regulate the structural stability of neurons.

Functional category	*De novo* variants	Familial variants
Cytoskeletal regulator	*CTTNBP2, ADNP, SYNGAP1*	*CTTNBP2, ADNP, SYNGAP1*
Adhesion molecule	***CDH10***, *CDH11, PCDH10, PCDH19, FAT1, CTNNA3, NRXN1-3, NLGN1*, ***NLGN2***, *NLGN3, CNTNAP2, CNTN4-6*	***CDH2, CDH8***, ***CDH9***, *CDH11*, ***PCDH9***, *PCDH10, PCDH19, FAT1, CTNNA3, NRXN1, NRXN2, NRXN3, NLGN1, NLGN3, CNTNAP2*, ***CNTN3***, *CNTN4, CNTN5-6*
Surface receptor	***GRIK4***, *NTRK3, GRIN2B*	***GRIK2***, *NTRK3, GRIN2B*
Signaling molecule	*DYRK1A, CDKL5, PTEN*	*DYRK1A, CDKL5, PTEN*
Synaptic molecule	*SHANK1-3, DLGAP2, STXBP5*	*SHANK1, SHANK2-3, DLGAP2, STXBP5*, ***PRICKLE1, CYFIP1***
Syndromic molecule	*FMR1, MECP2, UBE3A, TSC2*	*FMR1, MECP2, UBE3A*, ***TSC1****,TSC2*


*This table shows whether autism-risk variants found in these genes are *de novo* mutations or familial variants. Most genes have *de novo* and familial variants, however, some of genes show a preferential inheritance pattern (underlined; label in bold if only one pattern is found).*

Neuroanatomical studies of individuals with autism suggest a common disruption of neuronal structures with a decrease of dendrite arborization but an increase of dendritic spine density in select brain regions (Raymond et al., 1996; Bauman and Kemper, 2005; Hutsler and Zhang, 2010; Tang et al., 2014). This feature is distinct from other neurodevelopmental disorders, such as Rett or Fragile X syndromes, where the dendrite arbors and dendritic spine density are both downregulated (Kulkarni and Firestein, 2012). Intriguingly, dendrite arborization completes prior to dendritic spine formation during development. Although it has been proposed that the pruning mechanism of dendritic spines is defective in ASD (Frith, 2003), it is also plausible that the increase of dendritic spine density may be a compensation to re-establish the sufficient quantity of connections with fewer dendrite arbors. However, the precise spatial arborization of dendrites is critical for correct pre- and post-synaptic innervation when establishing the brain circuitry. The local increase of dendritic spine density may not be sufficient to compensate the effect from the loss of dendrite arbors, and may instead result in abnormal synaptic activity to disrupt normal neuronal function. This further emphasizes the importance of the establishment of structural integrity for neurons in order to provide proper brain function. In addition, the mechanisms of action of many current pharmacological agents for treating ASD affect normal neuronal function including the structural stability of neurons. With the early onset of ASD, the treatment often occurs at a very young age when the brain is still undergoing the period of development and maturation. As these pharmacological treatments may be beneficial to ameliorate some symptoms in ASD, the general brain development of these individuals may also be affected (Penagarikano, 2015). Thus, more precise circuitry-specific therapeutic intervention is needed to reduce the unwanted effect to the developing brain. Understanding the genetic and cellular pathways affected in ASD should provide more selective candidates for developing targeted intervention.

Although the list of autism-associated genes that regulate neuronal structures is extensive, it stands out that several genes actually function in the same signaling pathways (**Table 5**). For example, the mGluR5 pathway is disrupted when *Shank3* (Verpelli et al., 2011), *Fmr1* (Giuffrida et al., 2005; Dolen et al., 2007; Price et al., 2007; Wilson and Cox, 2007; Hays et al., 2011; Ronesi et al., 2012), *Ube3a* (Pignatelli et al., 2014), or *Mecp2* (Zhong et al., 2012) gene is altered. Application of mGluR5 antagonists has been shown to be promising to restore some phenotypes experimentally in neurons or animals with *Fmr1* mutants (Price et al., 2007; Wilson and Cox, 2007; Levenga et al., 2011b; Michalon et al., 2012, 2014; Ronesi et al., 2012; Pop et al., 2014). Whether targeting mGluR5 pathway can be clinically effective for ASD with mutations beyond *FMR1* will require further investigation. The mTOR pathway is defective when *Pten* (Jaworski et al., 2005; Kwon et al., 2006; Zhou et al., 2009; Pun et al., 2012), *Tsc1/2* (Gao et al., 2002; Meikle et al., 2008; Curatolo et al., 2016), or *Mecp2* (Ricciardi et al., 2011) gene is mutated. However, the mTOR pathway is upregulated in mice carrying defective gene products of *Pten. Tsc1/2*, or *Fmr1*, but downregulated in *Mecp2*-null mice. The mTOR inhibitor, rapamycin, has been shown to be effective to rescue some phenotypes caused by these mutations and has been used as a therapeutic agent to treat some of the autism symptoms (Meikle et al., 2008; Ehninger and Silva, 2011; Curatolo et al., 2016). The alteration of IGF-1/GSK3β pathway is implicated in *Pten* (Kwon et al., 2006), *Cdk5l* (Fuchs et al., 2014) or *Mecp2* (Itoh et al., 2007) mutant animals. Inhibition of GSK3β or application of IGF-1 can rescue the dendritic phenotype in *Cdk5l* and *Mecp2* mutant mice (Tropea et al., 2009; Fuchs et al., 2015; Della Sala et al., 2016). The mTOR pathway and the GSK3β pathway can be further linked together as they are both regulated by AKT. In addition, Rac1 activity is altered when *Elmo1* (Grimsley et al., 2004), *Cdk5l* (Chen et al., 2010b), *Shank3* (Duffney et al., 2015), or *Fmr1* (Lee et al., 2003; Chen et al., 2010a; Bongmba et al., 2011) is mutated suggesting its crucial role to maintain the stability of neurons. The potential therapeutic approach targeting the Rac1 pathway to rescue the neuronal and behavioral phenotype in mutant animals is actively under investigation (Hayashi et al., 2007; Duffney et al., 2015).

**Table 5:** The common signaling pathways that are altered by mutations of autism-risk genes.

Altered signaling pathway	Autism-risk gene involved	Available pharmacological agent
mGluR5	*SHANK3, FMR1, UBE3A, MECP2*	mGluR5 antagonists (e.g., fenobam, mavoglurant, CTEP, MPEP)
PI3K/Akt/mTOR	*PTEN, MECP2, TSC1/2*	mTOR inhibitor (e.g., rapamycin)
IGF-1/GSK3β	*PTEN, CDK5L, MECP2*	IGF-1, GSK3β inhibitor (e.g., SB216763)
Rac1	*ELMO1, CDK5L, SHANK3, FMR1*	N/A


*Several pharmacological agents targeting these pathways are available and have successfully rescued the neuronal phenotype and function experimentally. References can be found in the text.*

To date, several animal studies have tried to model the behavior phenotypes in autism, however, there remains a debate as to whether rodents can sufficiently recapitulate the complexities of the condition in human. The heterogeneity of genetic components also make it difficult to establish reliable animal models to describe the cellular and molecular mechanistic alterations in specific pathways. However, the studies on rodents can suggest which brain circuitry should be the area of interest for the corresponding behavior. iPSCs derived from ASD individuals appear to be an attractive model systems that allow researchers to directly investigate the interaction between the genetic contribution and the autism-relevant phenotypes. However, what is lacking in this system is a physiological relevant environment to correlate the behavior and the cellular phenotype. A recent emerging genetic editing technique, CRISPR/Cas9 (Jinek et al., 2012), is a powerful tool to study the mechanistic questions and identify the potential therapeutic interventions. Unlike the traditional knock-in or knock-out technique, CRISPR/Cas9 can introduce genomic editing of several genes at once. Using CRISPR/Cas9 in iPSCs can potentially determine the genetic contribution to the cellular phenotypes and provide a mechanism to correct them. However, there is still room for improvement of the efficiency and precision before this technique can be reliably used in clinical applications. Combining animal studies, iPSC models, and gene editing techniques, it is now possible to perform more comprehensive translational research in order to better understand the etiologies of ASD and design more efficient and effective therapeutic interventions.

## Author Contributions

All authors listed, have made substantial, direct and intellectual contribution to the work, and approved it for publication.

## Conflict of Interest Statement

The authors declare that the research was conducted in the absence of any commercial or financial relationships that could be construed as a potential conflict of interest.
